# Feasibility of non-linear beamforming ultrasound methods to characterize and size kidney stones

**DOI:** 10.1371/journal.pone.0203138

**Published:** 2018-08-28

**Authors:** Ryan S. Hsi, Siegfried G. Schlunk, Jaime E. Tierney, Kazuyuki Dei, Rebecca Jones, Mark George, Pranav Karve, Ravindra Duddu, Brett C. Byram

**Affiliations:** 1 Department of Urology, Vanderbilt University Medical Center, Nashville, Tennessee, United States of America; 2 Department of Biomedical Engineering, Vanderbilt University, Nashville, Tennessee, United States of America; 3 Department of Civil and Environmental Engineering, Vanderbilt University, Nashville, Tennessee, United States of America; The University of Hong Kong, HONG KONG

## Abstract

**Purpose:**

Ultrasound methods for kidney stone imaging suffer from poor sensitivity and size overestimation. The study objective was to demonstrate feasibility of non-linear ultrasound beamforming methods for stone imaging, including plane wave synthetic focusing (PWSF), short-lag spatial coherence (SLSC) imaging, mid-lag spatial coherence (MLSC) imaging with incoherent compounding, and aperture domain model image reconstruction (ADMIRE).

**Materials and methods:**

The ultrasound techniques were evaluated in an in vitro kidney stone model and in a pilot study of 5 human stone formers (n = 6 stones). Stone contrast, contrast-to-noise ratio (CNR), sizing, posterior shadow contrast, and shadow width sizing were compared among the different techniques and to B-mode. CT imaging within 60 days was considered the gold standard stone size. Paired t-tests using Bonferroni correction were performed to evaluate comparing each technique with B-mode.

**Results:**

Mean CT measured stone size was 6.0mm (range 2.9–12.2mm) with mean skin-to-stone distance 10.2cm (range 5.4–16.3cm). Compared to B-mode, stone contrast was best with ADMIRE (mean +12.2dB), while SLSC and MLSC showed statistically improved CNR. Sizing was best with ADMIRE (mean +1.3mm error), however this was not significantly improved over B-mode (+2.4mm). PWSF performed similarly to B-mode for stone contrast, CNR, SNR, and stone sizing. In the in vitro model, the shadow contrast was highest with ADMIRE (mean 10.5 dB vs 3.1 dB with B-mode). Shadow sizing was best with SLSC (mean error +0.9mm ± 2.9), however the difference compared to B-mode was not significant.

**Conclusions:**

The detection and sizing of stones are feasible with advanced beamforming methods with ultrasound. ADMIRE, SLSC, and MLSC hold promise for improving stone detection, shadow contrast, and sizing.

## Introduction

Kidney stones are highly and increasingly prevalent.[1] Diagnostic imaging is the primary means for the diagnosis, surveillance, and management of kidney stones.[2, 3] Ultrasound has several advantages over gold standard computerized tomography (CT) including its portability, accessibility, and avoidance of ionizing radiation exposure.[2] Among pediatric populations and pregnant women with kidney stones, several guideline panels recommend ultrasound as the first-line imaging modality for stone disease.[3–5] Despite the advantages with ultrasound, it suffers from poorer sensitivity (24–69%), diminished specificity (82–91%), and overestimation of stone size of approximately 2-3mm compared to CT. [6–12] It is not surprising that the role of ultrasound is currently limited to screening in the acute setting and surveillance.[2–4, 13–15] Improving the detection and sizing tasks would provide kidney stone patients more of the benefits inherent to ultrasound imaging.

Our group has been investigating several novel ultrasound imaging methods using advanced beamforming techniques that may hold promise for improving ultrasound’s capability to characterize kidney stones.[16] These include short-lag spatial coherence (SLSC) imaging, aperture domain model image reconstruction (ADMIRE), mid-lag spatial coherence (MLSC) imaging with incoherent compounding, and plane wave synthetic focusing (PWSF).[17–21] SLSC and ADMIRE are both non-linear ultrasound image formation methods that have both been shown to improve image quality that address the ubiquitous but understudied problem of reverberation and multipath scattering in clinical ultrasound. We developed MLSC—also a non-linear image formation method—specifically for improving ultrasound’s sensitivity to stones by enhancing coherent scatterers like stones, while suppressing the scattering from soft tissue.[16] In addition, we implement these methods in conjunction with synthetic aperture imaging. Specifically, we use angled plane wave transmit beams, which we refer to as plane wave synthetic focusing (PWSF), but in other literature, this is also referred to as plane wave compounding.[21] Here we use linear versus nonlinear in the mathematical sense to distinguish beamformers with nonlinear processing steps such as SLSC, ADMIRE and MLSC from beamformers such as delay and sum that utilize only linear processing.[22–25] The use of linear versus nonlinear in this manuscript does not imply anything about the physical acoustics of the imaging environment.

In our initial work in an in vitro model, we demonstrated that MLSC improved stone contrast and sizing compared to B-mode, while ADMIRE and SLSC also demonstrated improvements in sizing.[16] A limitation of the model was the lack of depths >8cm that would be expected for skin-to-stone distances in vivo. Therefore, the purpose of this study was to perform a feasibility study of advanced beamforming techniques in human stone formers to evaluate stone contrast and sizing error compared to gold standard CT. We also further investigate performance of these techniques for stone shadow contrast and shadow width sizing in vitro.

## Materials and methods

### In vivo study

#### Participants

**Fig 1:**
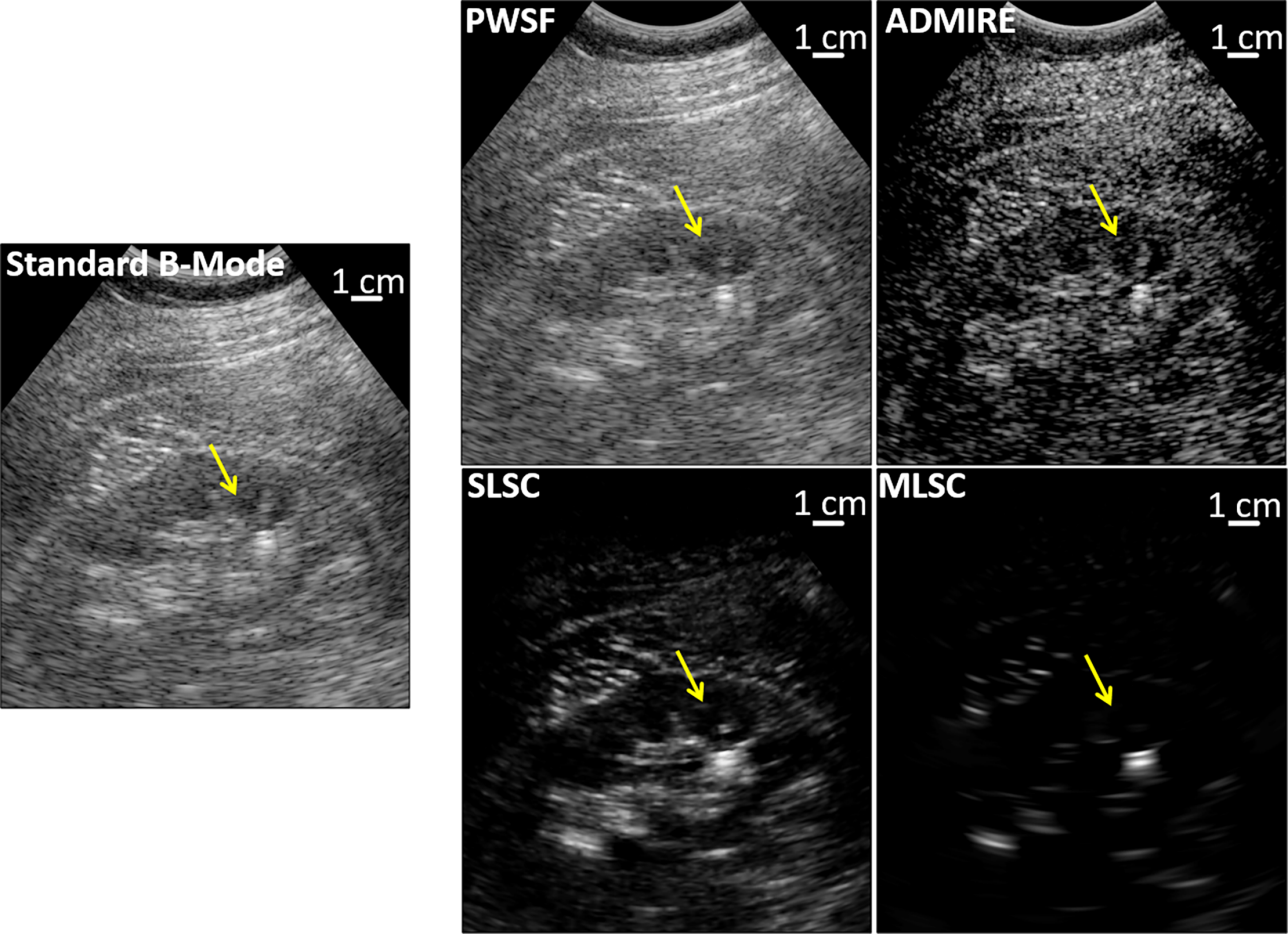
In vivo kidney stone case with standard B-mode and under the advanced beamforming methods. This patient had a CT scan showing a 14cm skin-to-stone distance, which is notable since depths >10cm are generally considered challenging with clinical ultrasound. PWSF is similar to B-mode except transmit focusing is performed everywhere in the image. Under ADMIRE, SLSC, and MLSC, the stone (yellow arrow) appears more echogenic. MLSC also suppresses the signal from the surrounding tissue.

We performed a prospective pilot study of kidney stone formers to investigate stone contrast and sizing error with standard B-mode, SLSC, ADMIRE, MLSC, and PWSF (Fig 1). The details of these methods have been previously described.[16] We recruited 5 kidney stone formers (Table 1) meeting the following inclusion and exclusion criteria: Inclusion criteria included CT imaging within 60 days demonstrating at least one ≥1mm renal stone measured in one dimension in one kidney. We limited the interval from CT to 60 days to minimize the potential for stone growth or spontaneous passage during this period. Exclusion criteria included vulnerable populations including children, incarceration status, pregnancy, inability to give informed consent, and serious illness likely to cause death within 5 years. The Vanderbilt University Institutional Review Board approved this study (IRB# 170001). Both written and verbal informed consent were obtained prior to enrollment into the study.

**Table 1:** Demographics and stone characteristics.

Subject	Age	Sex	Laterality	CT skin-stone distance (cm)	BMI (kg/m^2^)	CT measured size (mm)
1	60	M	Left	12.1	36.9	5.9
2	65	F	Right	16.3	42.3	6.3
3	74	F	Right	10.0	24.4	2.9
4	58	M	Right	5.4	22.6	4.2
5	51	F	Right	10.0	25.5	12.2
5	-	-	Left	7.5	25.5	4.7
**Mean**				10.2	29.5	6.0
**SD**				3.8	8.1	3.3

#### Data collection

During the study visit, participants underwent a renal ultrasound study using a research ultrasound system (Verasonics Vantage 128 system, Verasonics, Inc., Redmond, WA; C5-2 curvilinear probe). The Verasonics systems was used to acquire raw channel data from angled plane wave transmissions ranged between -30° and 30° spaced by 1° using a center frequency of 2MHz with a 1 cycle transmit pulse. The channel data were processed offline in MATLAB (Natick, MA) using the following beamforming methods: standard B-mode with a fixed transmit focus, SLSC, ADMIRE, MLSC, and PWSF (see S1 File and S1 Fig for descriptions for algorithms). We assumed a sound speed of 1540 m/sec through tissue.

Ultrasound images of each stone in the transverse (axial) and longitudinal (coronal) orientations were obtained by a physician unblinded to CT imaging to ensure the same stone was measured. Separately, the widths of the corresponding stones on clinical CT were measured in the respective orientations to determine size. The CT measurements were considered to be the gold standard.

#### Stone contrast

**Fig 2:**
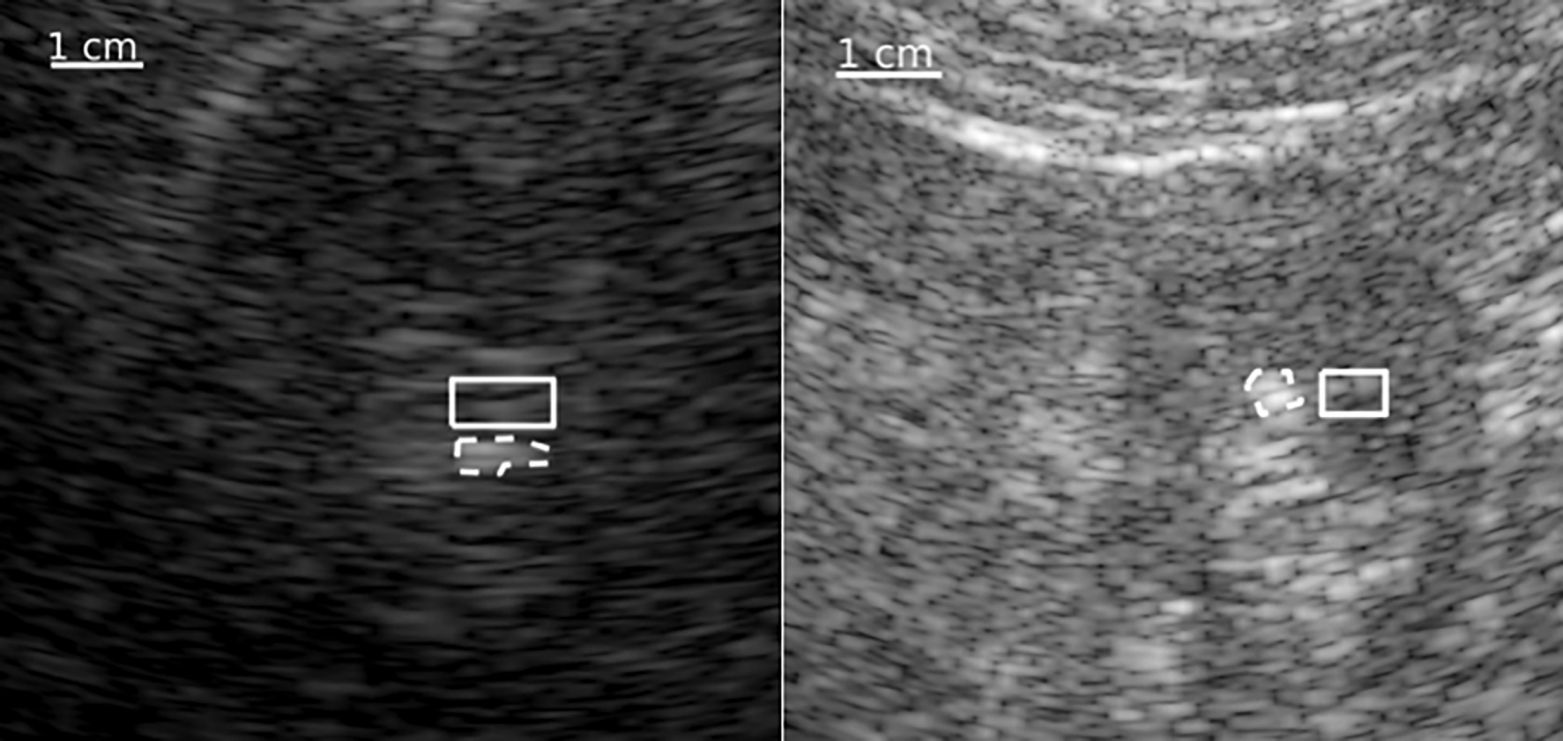
Region of interest (ROI) selection for stone and tissue background. Two examples are shown for selection of stone (dotted line) and background (solid rectangle).

The contrast of the stone with respect to the tissue background was calculated to determine how visible the stone was relative to the surroundings. The region of interest (ROI) of the background was selected near the same depth as the stone and of a similar size (ROI) (Fig 2). Both measurements were selected manually. This measurement is independent of machine post-processing algorithms such as gain. The stone contrast values were calculated using the following formula:

$$contrast_{stone}=20*\log_{10}\frac{\mu_{stone}}{\mu_{background}}$$

where μ is the mean intensity of the stone or background.

#### Stone sizing error

Accuracy of stone sizing was assessed by calculating the error (measurement error = ultrasound measured stone size–stone size on CT) for each measurement. The sizing error was calculated by subtracting the ultrasound measured size from the CT measured width in the same orientation. The ROI selection for the stone was performed similarly as described for stone contrast.

### In vitro study for posterior shadowing

#### Experimental setup and imaging protocol

Human calcium-based kidney stones were obtained during surgical extraction with ureteroscopy or percutaneous nephrolithotomy. Composition was determined with infrared spectroscopy to confirm calcium content. Excess stones meant to be discarded and without any patient identifiers were collected for this study.

**Fig 3:**
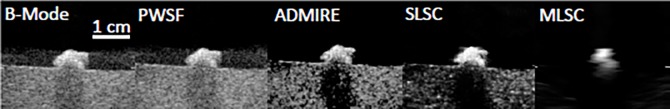
B-mode, PWSF, SLSC, MLSC, and ADMIRE images of a 10mm stone at 8cm depth. Note the posterior shadow appears below each of the stones.

All stones (n = 12, mean size 8.0 mm, range 2-18mm) were rehydrated and de-gassed at least 24hrs prior to imaging. Stones were placed on top of gelatin phantoms while immersed in a water bath. The gelatin phantoms were embedded with graphite to add diffuse scattering. The transducer (L7-4 linear array) was mounted above the stone and oriented to measure the maximum long axis length of the stone. Each stone was imaged at 8 cm using the Verasonics ultrasound system. Raw channel data were recorded from angled plane wave transmissions (-30° and 30° spaced by 1°) using a center frequency of 5.2MHz and a 1 cycle transmit pulse. We assumed a sound speed of 1480 m/sec. The channel data were processed offline as described previously with each of the beamforming methods (Fig 3).

#### Shadow contrast and sizing error

Similar to the stone contrast measurement, the shadow contrast was calculated using the following formula:

$$contrast_{shadow}=-20*\log_{10}\frac{\mu_{shadow}}{\mu_{gel}}$$

where μ is the mean intensity of the shadow or gelatin background. We introduced a negative into this realization of contrast so that higher contrast always signifies improvement.

**Fig 4:**
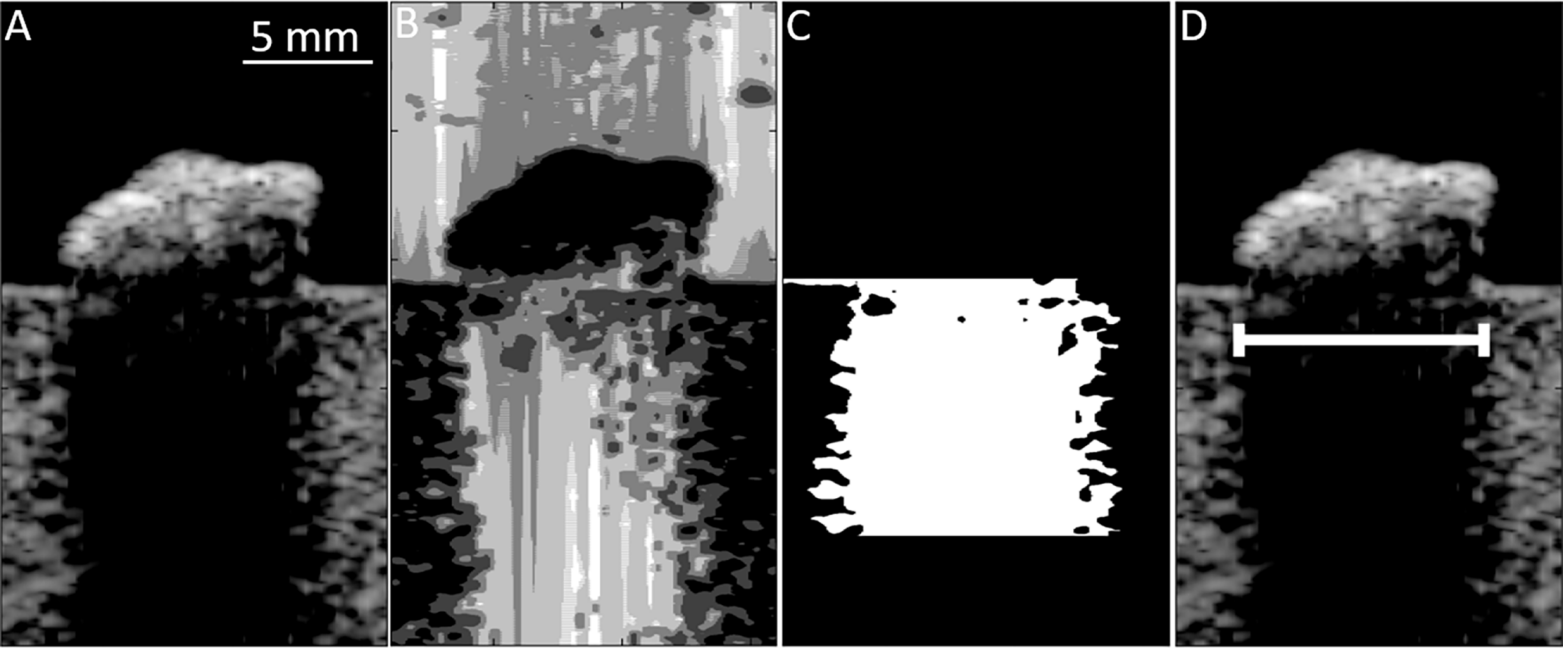
Schematic of automated algorithm to isolate shadow borders based on pixel intensity. A) Original processed image. B) Segmentation algorithm is applied. C) Shadow is selected based on segmentation and depth below stone (up to 1 cm). D) Lateral width is calculated as difference between the average edges of the shadow.

To provide an objective assessment of shadow width and minimize user bias, under each method the shadow borders in the ultrasound images were identified blinded to the CT measurement results and using an automated segmentation algorithm implemented in Matlab.[26] This method iteratively assigns all pixels in the image to a class based on the intensity of the pixel and those surrounding it (Fig 4). This initial segmentation requires minimal user input, which allows for greater consistency and accuracy compared to manual sizing methods. The lateral distance was determined as the difference between the average edge of the shadow on either side of the stone in the area 1 cm below the stone. Shadow sizing error was determined similarly by calculating the error (measurement error = ultrasound measured shadow width–manual measured stone width) for each measurement.

#### Statistical analysis

Contrast, contrast-to-noise ratio (CNR), signal-to-noise ratio (SNR), and sizing error values were compared among the different methods. Differences in contrast compared to B-mode were calculated by subtracting the contrast measurements. CNR was determined by the difference in the brightness of the stone to the background divided by the total variance of the background and stone:

$${CNR}_{stone}=20*\log_{10}\frac{\left|\mu_{stone}-\mu_{background}\right|}{\sqrt{\sigma_{stone}^{2}+\sigma_{background}^{2}}}$$

In other words, it is more indicative of the ability to detect meaningful change in contrast than the contrast metric alone. SNR was calculated as described by Smith et al. and indicates the detectability of the stone.[27] Repeated paired Student’s t-tests were used to analyze the differences of each group compared to B-mode, with a Bonferroni adjusted p<0.0125 considered significant. For sizing error, repeated paired Student’s t-tests were used to analyze the differences of each group compared to CT, with a Bonferroni adjusted p<0.01 considered significant.

## Results

Overall, six stones were imaged in five human subjects, with mean CT-measured stone size 6.0 mm (range 2.9–12.2mm) and skin-to-stone distance measured on CT 10.2cm (range 5.4–16.3cm) (Table 1).

**Fig 5:**
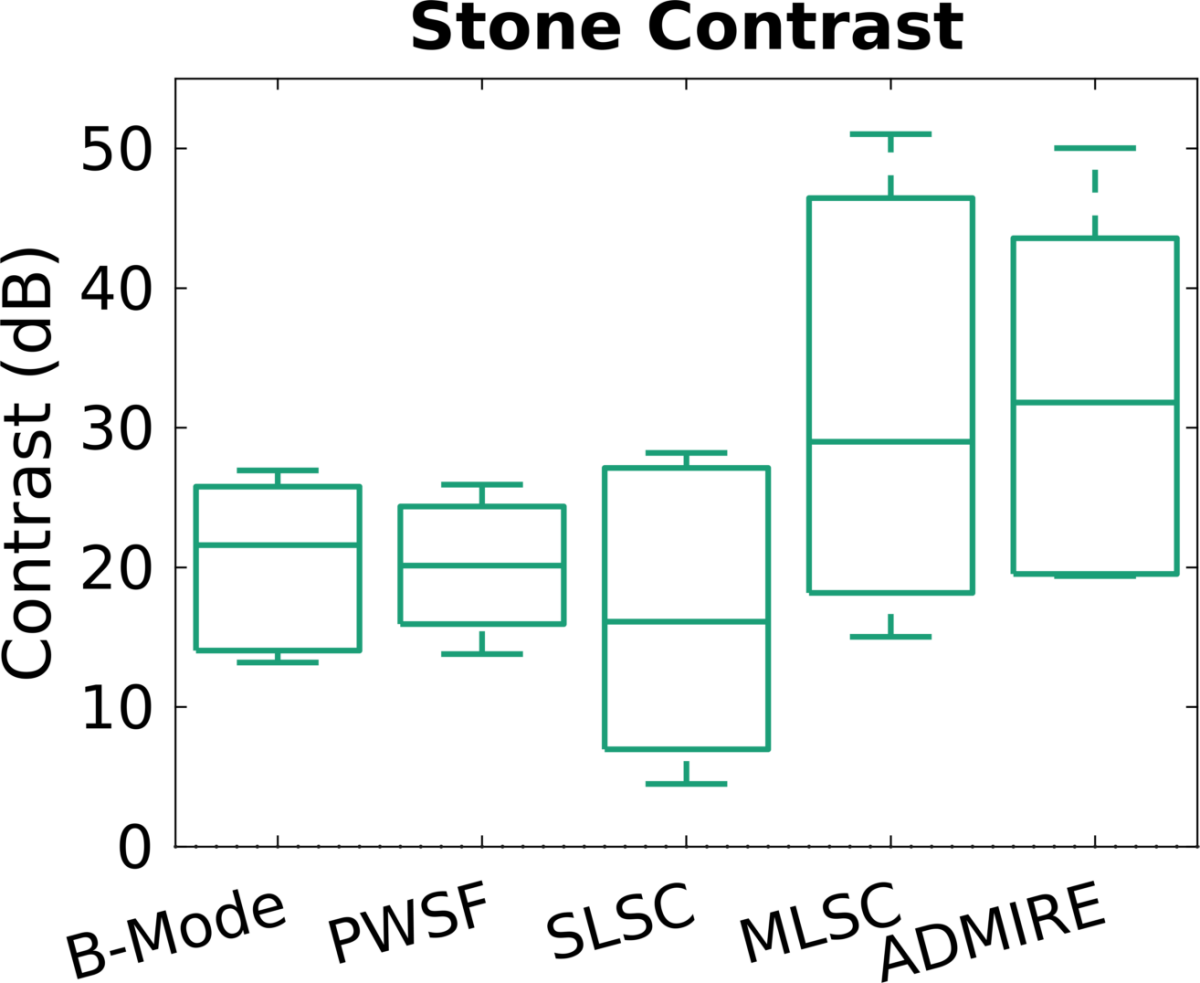
Stone contrast comparing each technique. Stone contrast (dB) is best with ADMIRE. SLSC and MLC also show improved SNR and CNR compared to B-mode.

**Fig 6:**
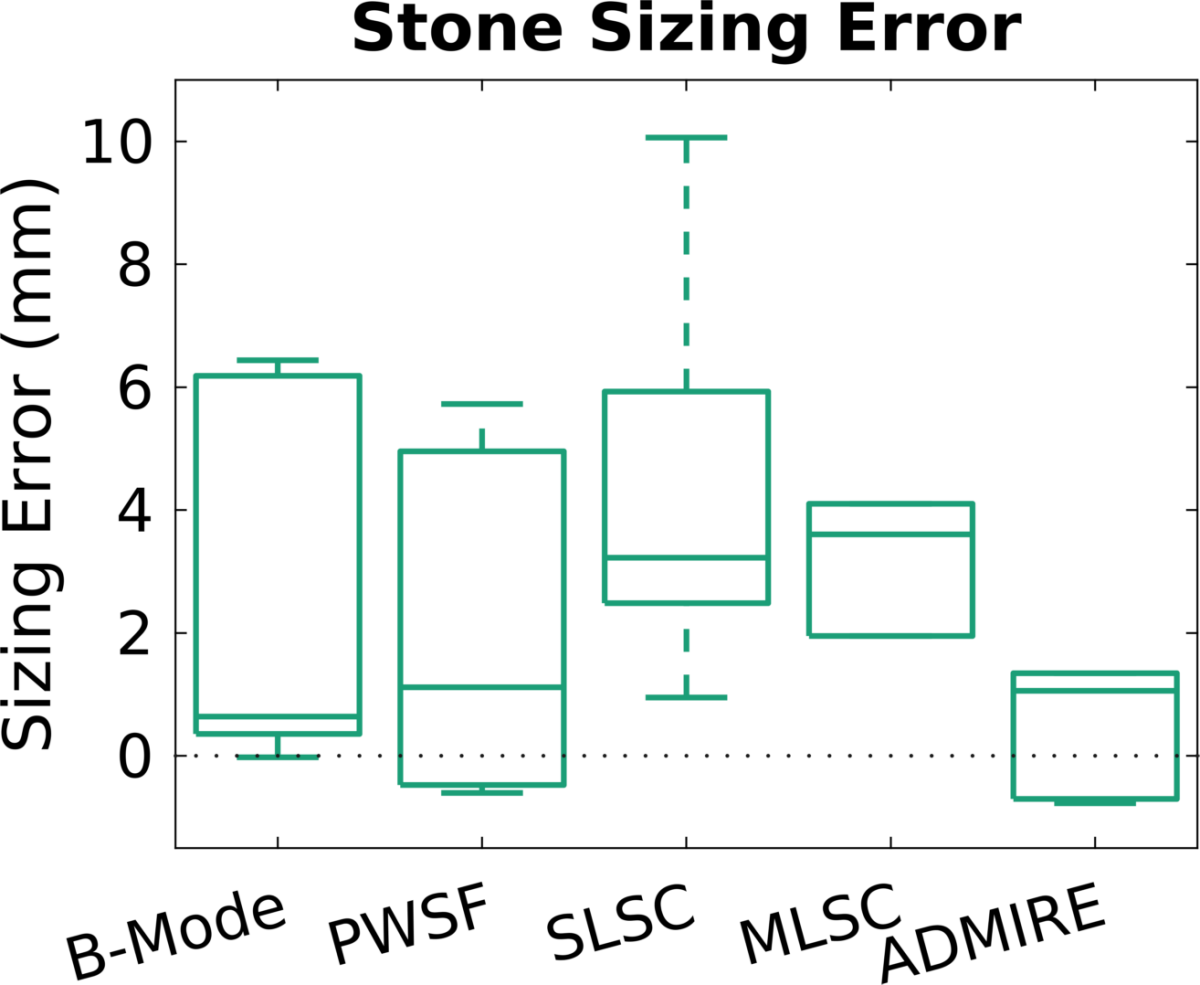
Stone sizing error using CT as the gold standard. ADMIRE had the least mean sizing error (mean +1.3mm error), but when compared to B-mode (mean +2.4mm error), the difference was not statistically significant.

For stone contrast (Table 2, Fig 5), ADMIRE (+12.2 dB, p = 0.010) was statistically better compared to B-mode for stone contrast. SLSC (p = 0.002) and MLSC (p = 0.016) were both statistically better compared to B-mode for stone CNR. SLSC (p<0.001) and MLSC (p<0.001) were both statistically better compared to B-mode for stone SNR. For stone sizing (Table 3, Fig 6), mean sizing error was best with ADMIRE (+1.3mm), however there was no statistically significant improvement when compared with B-mode (+2.4mm).

**Table 2:** Stone contrast performance with each technique relative to B-mode.

Relative to B-Mode		PWSF	SLSC	MLSC	ADMIRE
**Contrast (dB)**	**Mean**	-0.5	-4.0	11.3	12.2 ^a^
	**SD**	1.9	7.0	10.0	7.5
**CNR (dB)**	**Mean**	-0.4	5.9 ^b^	4.8 ^c^	-2.8
	**SD**	1.3	3.6	2.9	1.7
**SNR**	**Mean**	-0.2	5.6 ^d^	4.2 ^d^	0.1
	**SD**	0.5	3.0	1.7	0.5

^a^ ADMIRE vs B-mode, p = 0.010

^b^ SLSC vs B-mode, p = 0.002

^c^ MLSC vs B-mode, p = 0.016

^d^ vs B mode. P<0.001

**Table 3:** Sizing performance with each technique compared to CT.

Relative to CT*		B-Mode	PWSF	SLSC	MLSC	ADMIRE
**Sizing error (mm)**	**Mean**	2.4	2.0	4.3	3.8	1.3
	**SD**	3.1	2.7	3.3	4.1	2.5

*No significant differences seen among methods

**Fig 7:**
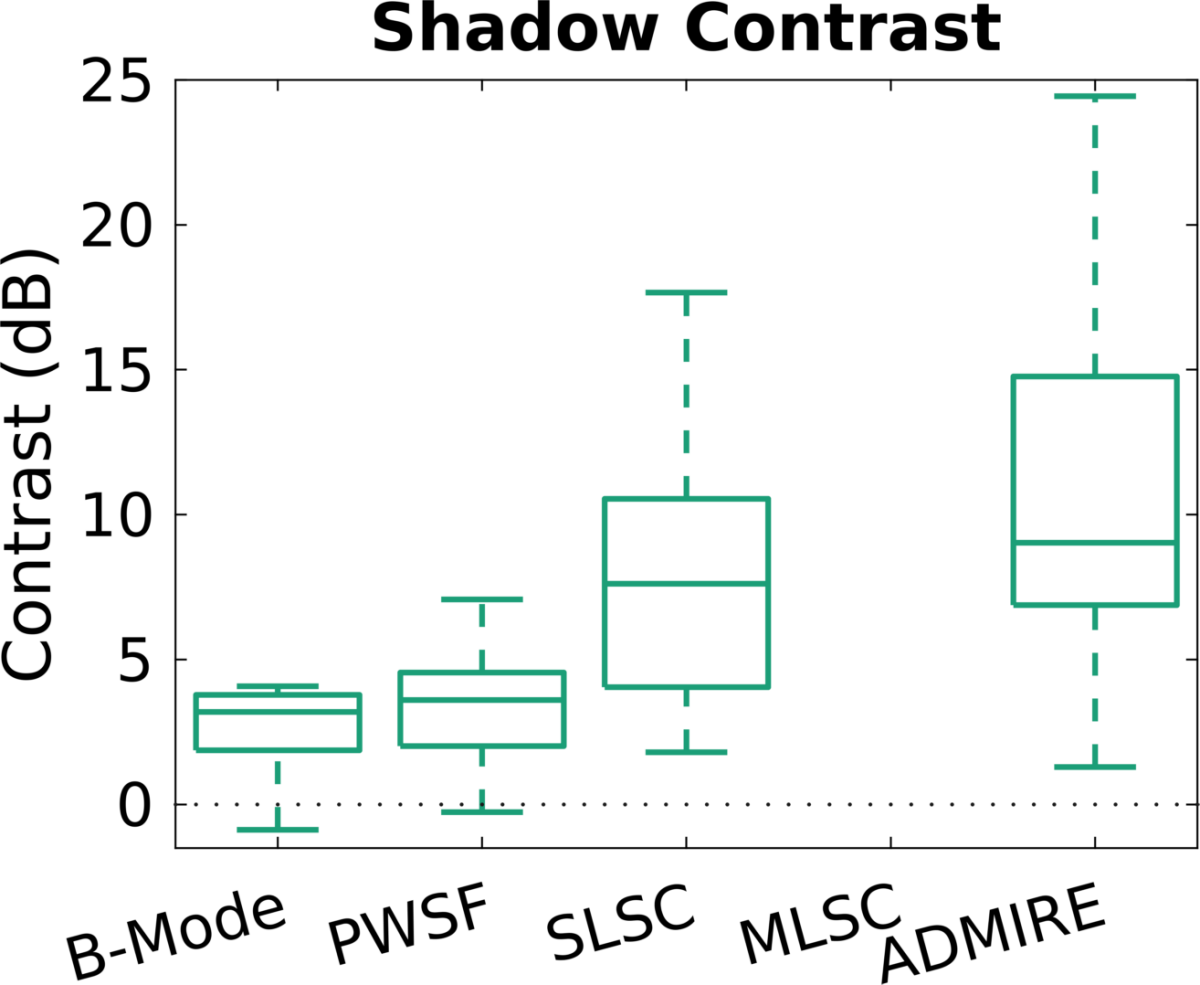
Shadow contrast comparing the different ultrasound methods. Contrast of the shadow (dB) was highest with ADMIRE. With MLSC, a shadow was not visible.

For posterior shadow contrast in the *in vitro* study, contrast was highest with ADMIRE, and significantly improved compared to B-mode (mean 10.5 dB vs 3.1 dB, respectively; p = <0.001) (Table 4, Fig 7). With MLSC, a shadow was not visible. Using the shadow to size the stone resulted in the least error with SLSC (mean error +0.9mm ± 2.9), however there were no significant differences seen comparing SLSC to B-mode (mean error -2.2mm ± 1.1). B-mode was the only method that was observed to be significantly different compared to the measured size of the stones (p = 0.010).

**Table 4:** Shadow contrast performance with each technique relative to B-mode.

Relative to B-mode		PWSF	SLSC	MLSC	ADMIRE
**Shadow contrast (dB)**	**Mean**	0.29	4.6*	n/a	7.4*
	**SD**	0.9	3.1		4.8

*SLSC (p<0.001) and ADMIRE (p<0.001) demonstrated significantly improved shadow contrast compared to B-mode.

## Discussion

We demonstrate the feasibility of advanced ultrasound beamforming for kidney stone contrast and sizing in this pilot study. Improvements in stone detection can, in part, be made by improving stone contrast relative to the surrounding tissue environment. In this study, stone contrast was highest with ADMIRE in the human stone formers, while CNR was best with SLSC and MLSC. These beamforming methods appear to address the kidney stone problem differently. ADMIRE models wavefronts from approximate points of origin to effectively suppress image degradation from bright scatterers within the tissue and multiple reflections from bright structures located near the transducer such as abdominal wall fascial layers. MLSC suppresses spurious points of coherence that occurs in the short lags thereby suppressing the echoes from the tissue environment. It should be worth noting that these methods are more than simply manipulating the “gain”, rather these are distinct methods from traditional delay-and-sum B-mode.

ADMIRE also holds promise to reduce sizing error from ultrasound, and more study is needed to determine whether there is a clinically meaningful benefit. It is reasonable that the ability to more sharply define the borders of the stone will reduce stone sizing error. Considering the typical stone-sizing error with B-mode of 2-3mm compared to non-contrast CT,[9, 11, 12] even small improvements in ultrasound performance may have significant clinical impact. Notably, often the decision to observe or perform surgical intervention is on the order of millimeters.

Adjuncts to standard B-mode have been introduced to improve standard B-mode ultrasound performance, but the impact has been modest. The detection of the “twinkling artifact” on color Doppler mode has been shown to increase detection sensitivity and specificity.[28–30] However, the clinical utility of this technique has been limited by the lack of twinkling in over 25% of stones and a high false-positive rate up to 60%.[28, 31] The presence of a shadow behind the stone, termed the “posterior acoustic shadow”, is associated with high specificity (95%) but poor sensitivity (31%).[31] While it has been shown to improve the sizing measurement closer to 1-mm error, a significant proportion of stones <5mm do not have a detectable shadow[32, 33] In our human pilot study, posterior acoustic shadowing was visible in 4 of 6 stones under a clinical ultrasound system, but was not visible under the research ultrasound system. The reasons for this are unclear and may include more sensitive transducers and more sophisticated B-mode post-processing for the clinical scanner. ADMIRE appeared to demonstrate the best shadow contrast in vitro, however this finding would need to be replicated in a larger population human stone formers.

A separate confounding factor to stone imaging is the sound speed mismatch. In general sound speed inhomogeneity is known to induce degradation in ultrasound imaging. The role of sound speed has not been studied specifically for stones, but in general the faster speed of sound in stones—about 33% greater than tissue—will result in an underestimation of the axial length. In the lateral dimension the speed of sound will induce a slightly wider measurement, which has been characterized for delay and sum beamforming generally and more recently in ADMIRE.[34–36] It is less clear how this sound speed mismatch will impact SLSC or MLSC because this has not been characterized. Finally, the role of sound speed in interfering with the lateral sizing is related to the integrated sound speed along the propagation path. Because the stones are generally fairly small, this will work to mitigate the effect of stone sound speed on sizing error.

In this work we implemented SLSC, ADMIRE, and MLSC methods in conjunction with PWSF, which is a synthetic transmit focusing method. Earlier works have indicated that PWSF can enhance both SLSC and ADMIRE.[18, 37] With SLSC, synthetic aperture methods are known to increase the depth of field. With ADMIRE, synthetic aperture methods produce a more uniform speckle texture throughout the image. However, both of these methods would produce similar performance with a fixed focus transmit beam if the focus was within the kidney. MLSC was also implemented with plane waves but not with PWSF. Implementing MLSC with plane waves is a natural choice because MLSC requires an incoherent transmit beam. MLSC could also be implemented with a fixed focus beam, but in this case it would be important to position the focus well outside the kidney. This would ensure that the transmit beam is relatively incoherent within the kidney itself.

These advanced beamforming methods were each designed to address image degradation mechanisms inherent to ultrasound when applied to the complex heterogeneous environment found during in vivo imaging. These mechanisms include multiple scattering, bright off-axis scattering, phase aberration and gross sound speed mismatch.[38–49] Therefore, because these methods were developed to address challenging imaging environments, one would expect that the performance of these methods would be robust to clinical challenges such as increased skin to stone distance (e.g. central obesity), reverberation from the abdominal wall and other structures, and complex stone geometries (e.g. other than smooth/round). In addition, primary goal in this work was to evaluate in vivo feasibility so the methods compared here were implemented in Matlab without a significant concern for efficiency. However, the speed of these methods have been considered elsewhere and in many cases real time implementations have already been developed.[50–52] In this case we assume that real-time implementations of SLSC will be consistent with real-time implementations of MLSC, but because of the order of operations MLSC will always be slightly slower. Future work is needed to refine roles of different beamforming methods for specific kidney stone imaging tasks and validation of these results in a more robust clinical study.

## Conclusions

The advanced ultrasound beamforming methods ADMIRE, MLSC, and SLSC appear to improve kidney stone contrast compared to standard B-mode ultrasound. ADMIRE also holds promise for reducing stone sizing error and enhancing the detection of the posterior acoustic shadow. These technologies may enable broader adoption of ultrasound methods for kidney stone care, and further study is needed to refine and validate these techniques.

## Supporting information

S1 File Detailed descriptions of the ultrasound beamforming methods. (DOCX)

S1 Fig Schematic of each of our advanced beamforming methods. The methods start by transmitting incoherent beams at various angles. Plane waves are shown here as an example of an incoherent beam. Delays are applied. Then, the transmissions are summed and processed to create an ultrasound image. **PWSF** achieves transmit focusing at all depths instead of at just a single depth as in standard B-mode. **ADMIRE** is a model-based beamforming approach that explicitly integrates physics into B-mode image formation. **MLSC** is sensitive to only intrinsic tissue coherence from objects like stones and suppresses other features including most tissue. **SLSC** creates images correlated to the phase of the ultrasound wavefronts across the transducer surface, as compared to B-mode where images are sensitive to amplitude.
(TIF)

S2 File Abbreviations used. (DOCX)
