# Supplementary material for: Feasibility of non-linear beamforming ultrasound methods to characterize and size kidney stones
Source: PLoS One. 2018 Aug 28;13(8):e0203138. doi: 10.1371/journal.pone.0203138 (PMC6112662; doi:10.1371/journal.pone.0203138)
Supplement: S1 File — (DOCX) [file pone.0203138.s001.docx]

**Ultrasound beamforming methods**

The raw channel data in this study were processed using the following beamforming methods:

**1. Conventional B-mode (B-mode)**

Conventional beamforming applies delays to each transmitted channel signal to focus at a single depth and lateral location. The received signals are then dynamically delayed to achieve receive focusing at all depths for that lateral location. These channel signals are summed to generate a single line of the image. This process is repeated for multiple lateral locations to generate a full image. Here, the overall process has been also referred to as delay-and-sum beamforming.

**2. Plane Wave Synthetic Focusing (PWSF)^21^**

Transmit synthetic aperture focusing enables ultrasound systems to create synthetic transmit focuses throughout the image much like modern systems all utilize dynamic receive focusing to focus everywhere on the receive signal. Many different transmit synthetic aperture techniques are available, but here we implemented a scheme that relies on transmitting plane waves at different angles which we refer to as plane wave synthetic focusing (PWSF). It is akin to conventional B-mode imaging except that it achieves transmit focusing at all depths instead of at just a single depth. For the *in vitro* study, PWSF was implemented with a linear array transducer and true plane waves. For the *in vivo* study, PWSF was implemented on a curvilinear array transducer. Therefore, the beams had a curvature matching the shape of the curvilinear array.

**3. Short-lag spatial coherence (SLSC)^17, 18^**

This method creates images correlated to the coherence of the ultrasound wavefronts across the surface of the transducer. This coherence metric can be calculated from the transmit beamformed, receive delayed but unsummed ultrasound channel data. The channel data are windowed to preserve axial resolution, and then each window is multiplied by the other channels and then normalized by the signal energy within the channels in order to create a measure of coherence. The coherence across nearby element spacings is summed to create each pixel in the final image. The original implementation of SLSC had a limited depth of field so we implemented SLSC with PWSF. SLSC has been shown to improve image quality in a range of scenarios, but is particularly suited to imaging difficult to image patients where image degradation is known to be a problem. We implemented SLSC with lags 1-10 with each lag averaged across the array.

**4. Mid-lag spatial coherence (MLSC) with incoherent compounding^16^**

This method is similar to SLSC but the order of the processing steps is rearranged to preferentially suppress the signal from tissue. To this end, MLSC performs the spatial coherence quantification used with SLSC before transmit beamforming. This means that there is no introduction of spatial coherence by the transmit beamforming as occurs in SLSC. Similar to SLSC, MLSC was implemented using plane waves but the plane waves were not summed coherently as with PWSF. The coherence images from each angled plane wave are summed together in order to enhance the stone and suppress any spurious correlations in the tissue. Additionally, because spurious points of coherence that may occur will occur in the shortest lags, these are excluded from the sum used to create the coherence image; hence, the reference in the name to mid-lag. We implemented MLSC using lags 3-10 with each lag averaged across the array.

**5. Aperture domain model image reconstruction (ADMIRE)^19, 20^**

ADMIRE is an explicit model-based method. The physics of linear ultrasound wave propagation are well known, and the effect of various sources of image degradation such as bright sources (i.e. stones) or reverberant sources such as those coming from shallow fat and muscle tissue can easily be modeled. By modeling a large number of these sources, the ultrasound wavefront returning to the transducer at a given time can be broken down into approximate points of origin. Then, ultrasound pressure waves originating only from a given region of interest can be reconstructed into a high-quality B-Mode signal. Like SLSC or MLSC, ADMIRE processing is applied to each pixel in the image, and ADMIRE was implemented here in conjunction with PWSF.
