# Supplementary figures and images for: Feasibility of non-linear beamforming ultrasound methods to characterize and size kidney stones
Source: PLoS One. 2018 Aug 28;13(8):e0203138. doi: 10.1371/journal.pone.0203138 (PMC6112662; doi:10.1371/journal.pone.0203138)

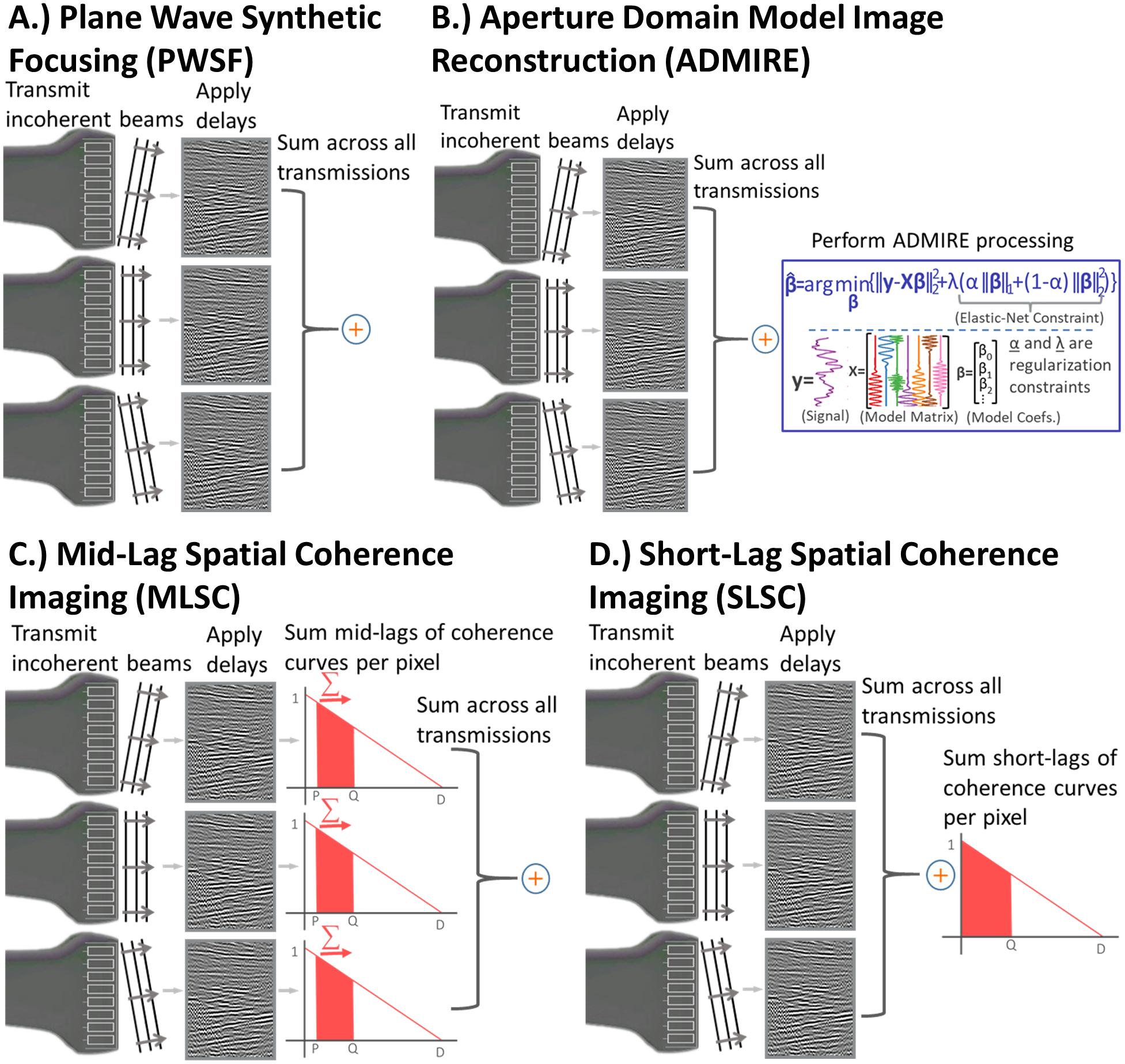

Supplement: S1 Fig — The methods start by transmitting incoherent beams at various angles. Plane waves are shown here as an example of an incoherent beam. Delays are applied. Then, the transmissions are summed and processed to create an ultrasound image. PWSF achieves transmit focusing at all depths instead of at just a single depth as in standard B-mode. ADMIRE is a model-based beamforming approach that explicitly integrates physics into B-mode image formation. MLSC is sensitive to only intrinsic tissue coherence from objects like stones and suppresses other features including most tissue. SLSC creates images correlated to the phase of the ultrasound wavefronts across the transducer surface, as compared to B-mode where images are sensitive to amplitude. (TIF) [file pone.0203138.s002.tif]
