# Supplementary material for: Feasibility of non-linear beamforming ultrasound methods to characterize and size kidney stones
Source: PLoS One. 2018 Aug 28;13(8):e0203138. doi: 10.1371/journal.pone.0203138 (PMC6112662; doi:10.1371/journal.pone.0203138)
Supplement: S2 File — (DOCX) [file pone.0203138.s003.docx]

**Abbreviations Used**

ADMIRE = aperture domain model image reconstruction

CNR = contrast-to-noise ratio

CT = computed tomography

MLSC = mid-lag spatial coherence

PWSF = plane wave synthetic focusing

SLSC = short-lag spatial coherence

SNR = signal-to-noise ratio
